# Automated classification of skeletal malocclusion in German orthodontic patients

**DOI:** 10.1007/s00784-025-06485-0

**Published:** 2025-08-05

**Authors:** Eva Paddenberg-Schubert, Kareem Midlej, Sebastian Krohn, Erika Kuchler, Nezar Watted, Peter Proff, Fuad A. Iraqi

**Affiliations:** 1 Department of Orthodontics, University Hospital of Regensburg, University of Regensburg, 93047 Regensburg, Germany; 2 Department of Clinical Microbiology and Immunology, Faculty of Medicine and Health Sciences, Tel Aviv University, Tel Aviv, 6997801 Israel; 3 Department of Orthodontics, University of Bonn, D-53111 Bonn, Germany; 4 Center for Dentistry Research and Aesthetics, Jatt, 4491800 Israel; 5 Department of Orthodontics, Faculty of Dentistry, Arab American University, Jenin, PNA Palestine; 6 Gathering for Prosperity Initiative, Jatt, 4491800 Israel

**Keywords:** Skeletal malocclusion, Artificial intelligence, Orthodontic diagnostics, Cephalometric analysis, Individualized ANB, Orthodontic treatment planning

## Abstract

**Objectives:**

Precisely diagnosing skeletal class is mandatory for correct orthodontic treatment. Artificial intelligence (AI) could increase efficiency during diagnostics and contribute to automated workflows. So far, no AI-driven process can differentiate between skeletal classes I, II, and III in German orthodontic patients. This prospective cross-sectional study aimed to develop machine- and deep-learning models for diagnosing their skeletal class based on the gold-standard individualised ANB of Panagiotidis and Witt.

**Materials and methods:**

Orthodontic patients treated in Germany contributed to the study population. Pre-treatment cephalometric parameters, sex, and age served as input variables. Machine-learning models performed were linear discriminant analysis (LDA), random forest (RF), decision tree (DT), K-nearest neighbours (KNN), support vector machine (SVM), Gaussian naïve Bayes (NB), and multi class logistic regression (MCLR). Furthermore, an artificial neural network (ANN) was conducted.

**Results:**

1277 German patients presented skeletal class I (48.79%), II (27.56%) and III (23.64%). The best machine-learning model, which considered all input parameters, was RF with 100% accuracy, with Calculated_ANB being the most important (0.429). The model with Calculated_ANB only achieved 100% accuracy (KNN), but ANB alone was inappropriate (71–76% accuracy). The ANN with all parameters and Calculated_ANB achieved 95.31% and 100% validation-accuracy, respectively.

**Conclusions:**

Machine- and deep-learning methods can correctly determine an individual’s skeletal class. Calculated_ANB was the most important among all input parameters, which, therefore, requires precise determination.

**Clinical relevance:**

The AI methods introduced may help to establish digital and automated workflows in cephalometric diagnostics, which could contribute to the relief of the orthodontic practitioner.

**Supplementary Information:**

The online version contains supplementary material available at 10.1007/s00784-025-06485-0.

## Introduction

Within the German population growing patients and adults present a high demand and need for orthodontic treatment to correct various types of malocclusion or dysgnathia [1, 2]. Prior to the actual orthodontic treatment, precise diagnoses are mandatory to allow for personalised treatment plans [3]. Therefore, various diagnostic procedures are necessary, often including, among others, lateral cephalograms and their analyses, which are comprised of skeletal, dental and soft-tissue parameters in the sagittal and vertical dimension.

One of the skeletal sagittal variables of high importance is skeletal class, describing the antero-posterior relation of the upper and lower jaw base. There is a variety of possibilities to determine an individual’s skeletal class. Although the method of measuring the ANB angle, introduced by Riedel [4], is well established and used by many orthodontists, this procedure bears the risk of distortion of the true skeletal class due to geometric dependencies of the anatomic structures concerned and the corresponding reference points [5]. Hence, both different cephalometric parameters and various individualisation methods, including graphical solutions and regression equations, have been developed to increase the precision in defining the true sagittal relation of the maxilla and mandible of the individual patient [5–8]. The advantage of individualising cephalometric norm values for each patient is the increased precision in diagnosis and therefore in treatment planning. Within this context, one frequently used regression equation is the individualised ANB, which was introduced by Panagiotidis and Witt [7]. Their method was updated and optimised by Paddenberg and Kirschneck and will be referred to as iANB_KP in this manuscript [5]. Another well spread technique applied to determine a patient’s skeletal class, which will be evaluated in this study, is the Wits appraisal, which was established by Jacobson [6].

In orthodontics, as well as in other fields of dentistry, artificial intelligence (AI) is of increasing interest and steadily improves [9–11]. Frequent applications of AI in orthodontics include, for example, the automated identification of cephalometric landmarks [12, 13]. Other implications include the prediction of the duration of orthodontic treatment [14] or assist the practitioner in treatment decisions, such as with extractions of teeth [15]. However, many AI systems still require further improvement and cannot substitute the orthodontist yet [15]. So far, various AI-models have been described to conduct cephalometric analyses [16, 17]. Some studies specifically focus on the automated determination of patients’ skeletal class. For example, Nan et al. established a deep-learning method in Chinese children for the classification of skeletal class I, II and III, and validated it by comparing it to their gold standard, which was a combination of ANB and Wits appraisal [18]. Ueda et al. evaluated, next to vertical parameters, the performance of various AI-models for the determination skeletal class based on ANB [19]. They considered only Japanese adults in their analysis. Midlej et al. assessed various machine-learning models to correctly classify Arab patients as skeletal class II and III [20] or as skeletal class I and II [21].

However, to the best of our knowledge there is no study available, which evaluates machine-and deep-learning models to precisely diagnose the skeletal class of German orthodontic patients of all ages. Hence, the main aim of this prospective cross-sectional study was the development and assessment of the performance of machine- and deep-learning models to correctly differentiate between skeletal class I, II and III in German orthodontic patients of all ages more accurately compared to the gold standard individualised ANB [7].

## Materials and methods

### Cephalometric parameters

In this study, 24 cephalometric parameters as well as the covariates sex and age were included in the analysis. The cephalometric parameters included in this study are summarised in Supplementary Tables 1 and Supplementary Fig. 1.

### Inclusion and exclusion

Patients, receiving orthodontic treatment between 03/2023 and 12/2024 were screened for inclusion. All patients of the study centres were considered for inclusion, irrespective of their malocclusion, sex or age.

#### Inclusion criteria

1. Availability of pre-treatment lateral cephalograms with scale.
2. Skeletal class I, II or III according to the Calculated_ANB, which is defined as ANB angle- individualised ANB of Panagiotidis and Witt [7]:
  Skeletal class I (−1.5 < Calculated_ANB < 1.5).
  Skeletal class II (Calculated_ANB > 1.5).
  Skeletal class III (Calculated_ANB<−1.5).

3. Demographic data including information about sex and age.

#### Exclusion criteria

1. Patients/guardians who were not able or refused to sign an informed consent form.
2. Missing pre-treatment lateral cephalogram with scale and/or information about sex and age.

### Data analysis

High-resolution cephalograms of each patient were imported without loss of resolution using TIF format into Ivoris^®^ analyze pro (Computer konkret AG, Falkenstein, Germany; version 8.2.83.130). Precise calibration was performed within the software environment prior to analysis to ensure metric accuracy. Prior to the data analysis, interrater- and intrarater - reliability of the cephalometric analysis was tested by validating 50 randomly chosen lateral cephalograms, which were evaluated twice by two different raters (SK, EP). Besides, the same rater evaluated the images with interval of minimum two weeks. Data analysis was applied with Python software using the Google Colab platform [22]. In this study, different classification algorithms, including linear discriminant analysis (LDA), random forest (RF), decision tree (DT), K-nearest neighbors (KNN), support vector machine (SVM), and Gaussian naïve Bayes (NB), and multi-class logistic regression (MCLR) were used. All these machine-learning models were applied using the Scikit-Learn Python package [23]. Besides, a deep-learning model, more precisely an artificial neural network (ANN) was performed to classify the patients as skeletal class I, II, or III patterns based on the cephalometric parameters and the covariates sex and age. The ANN provides a means for dealing with complex pattern-oriented problems of categorisation and time-series (trend analysis) types [24].

Further analyses consisted of a comparison between different classification methods, i.e., between the gold standard Calculated_ANB and the alternative methods Wits appraisal [6] and iANB_KP [5], by a confusion matrix and Cohen’s Kappa. Finally, Wits appraisal was used to re-classify the study population and repeat both the machine- and deep-learning methods.

### Sample size

This study enrolled the maximum available cases within the period of patient enrolment (03/2023-12/2024). Besides, the current sample size achieved the expected accuracy results on the validation data (20% of the data).

### Data preprocessing

In the current study, the downsampling process was used to balance the classification groups, followed by the Python function- Random UnderSampler, where samples from the targeted classes are removed randomly [25]. In addition, due to the different scales of the parameters and variables, data was standardised before applying the models.

### Machine-learning algorithms

For each model, based on the random test data that was selected using Python software code, that ensured reproducibility by using a fixed random seed (20% unseen data), we calculated the accuracy, as well as the precision, recall and F1 score. Accuracy determines the numbers in accordance with the actual and the predicted skeletal class using the complete data set. The F1 score evaluates the accuracy of a machine-learning model, but, contrary to accuracy, it focuses on the accuracy for each class (skeletal class I, II, III), which is of advantage in case of unequal sizes of the classes. Calculation of the F1 score is based on the precision and recall. Precision is also called positive predictive value, and recall expresses the sensitivity of the machine-learning model. Furthermore, the importance of the input parameters was determined and used to develop machine-learning models based on the most important variables only.

### Applying both machine learning & deep learning models

In this study, we aimed to apply both machine learning and deep learning models. Machine learning needs algorithms, learn from that data and then classify the data, while deep learning consists of many hidden layers and multiple neurons per layer. Besides, deep learning takes a large amount of data while machine learning needs a small amount of data to work and arrive at a conclusion [26]. In this study we used both tools (i.e., machine learning and deep learning) to examine the ability of each tool to classify patients. Although this study demonstrated the ability of machine learning models to classify patients with very high accuracy, we also applied deep learning as an introduction to a general model that will include more data from our population and from other populations. In other words, the deep learning models introduced in this study will be a basis for more general and complex models in further studies.

## Results

This study consisted of the coded data of 1277 German patients diagnosed as skeletal class I (*n* = 623, 48.79%), II (*n* = 352, 27.56%) or III (*n* = 302, 23.64%). Both interrater (0.92 to 0.99) and intrarater reliability (0.90 to 0.99) were almost perfect as required for subsequent data analysis. Among skeletal class I patients, mean age was 13 (M = 13, SD = 3.8), and most were females (*n* = 341, 54.73%). The mean age of skeletal class II patients was also 13 (M = 13, SD = 6), and 56.53% (*n* = 199, 56.53%) were females. Finally, among skeletal class III patients, the mean age was 14 (M = 14, SD = 5.6), and here also, 53.64% were females (*n* = 162, 53.64%). The full results of the demographic variables and the cephalometric parameters descriptive statistics are detailed in Table 1. After the downsampling process, the sample size consisted of 956 patients- 302 skeletal class I patients, 352 skeletal class II patients, and 302 skeletal class III patients. 80% (*n* = 764) of the total study population after the downsampling were used as a training set to develop the AI-models.

**Table 1:** Descriptive statistics of the skeletal and dental cephalometric parameters for skeletal class I, II, and III - sample size (N), mean, and standard deviation

Skeletal class	I			II			III		
Variable	*N*	Mean	SD	*N*	Mean	SD	*N*	Mean	SD
Age	623	13	3.8	352	13	6	302	14	5.6
Sex - Females	341 (54.73%)			199 (56.53%)			162 (53.64%)		
NL/ML	623	24	5.9	352	22	5.7	302	24	5.6
NL/NSL	623	7.4	3.4	352	8.4	3.2	302	6.9	3.2
PFH/AFH	623	67	5	352	67	5.1	302	67	5.1
Gonial angle	623	122	6.2	352	119	6.7	302	125	6.5
Facial axis	623	90	4.3	352	89	4.1	302	92	4.5
SNA angle	623	81	3.7	352	81	3.4	302	81	3.8
SNB angle	623	78	3.1	352	75	2.9	302	81	3.5
ANB angle	623	3.6	1.6	352	6.2	1.7	302	0.42	2.1
ANBind	623	3.6	1.4	352	3.5	1.5	302	3.5	1.5
Calculated_ANB	623	0.019	0.84	352	2.8	1	302	−3.1	1.6
SN-Ba	623	132	4.9	352	133	4.6	302	130	5.2
SNP-g	623	79	3.2	352	76	3	302	82	3.6
SN (mm)	623	66	3.9	352	66	3.9	302	66	3.9
GoMe (mm)	623	67	5.1	352	65	5.2	302	69	5.8
Wits	623	0.12	2.2	352	3.5	2.3	302	−3.9	2.8
ML-NSL	623	31	5.9	352	31	5.9	302	31	6
+ 1/NL angle	623	68	8.3	352	71	10	302	65	7.1
+ 1/SNL angle	623	76	8.5	352	79	11	302	72	7.3
+ 1/NA angle	623	23	8.3	352	20	11	302	27	6.8
+ 1/NA (mm)	623	3.7	2.7	352	2.2	3.3	302	5	2.4
−1/ML	623	84	6.7	352	80	7.3	302	89	7.4
−1/NB angle	623	25	6.9	352	26	7.6	302	22	7.1
−1/NB (mm)	623	3.8	2.4	352	4.1	2.4	302	2.9	2.3
Interincisal angle	623	128	12	352	128	14	302	130	11

### General machine-learning models

The general machine-learning model included 24 cephalometric parameters as well as sex and age as covariates. Among the seven machine-learning models performed, random forest was the most accurate to classify the patients as skeletal I, II or III. In this model, the results showed 100% accuracy. The model showed 100% precision, recall and F1 scores in each class. Concerning the other models, the results showed 99% accuracy in the decision tree, 98% accuracy in the support vector machine and multi-class logistic regression models, 93% accuracy in the linear discriminant analysis model, 91% accuracy in the Gaussian naïve Bayes model and 85% accuracy in the k-nearest neighbours model. The detailed results are presented in Table 2.

**Table 2:** Performance of different machine-learning models in predicting skeletal class I, II, and III - Linear discriminant analysis, random forest, decision tree, K-nearest neighbours, support vector machine, Gaussian Naïve bayes, and multi-class logistic regression. For every model, precision, recall, F1 score, and accuracy scores are presented

		Included parameters: 24 cephalometries & 2 covariates			Included parameters: Calculated_ANB (ANB – ANB_ind_)			Included parameters: SNA, SNB & ML-NSL angles		
	Class	Precision	Recall	F1score	Precision	Recall	F1score	Precision	Recall	F1score
Linear Discriminant Analysis	I	0.87	0.91	0.89	0.98	0.96	0.97	0.96	0.96	0.96
	II	0.99	0.95	0.97	0.97	1.00	0.99	0.97	1.00	0.99
	III	0.93	0.93	0.93	1.00	0.98	0.99	1.00	0.97	0.98
Accuracy				0.93			0.98			0.98
Random Forest	I	1.00	1.00	1.00	-	-	-	1.00	0.98	0.99
	II	1.00	1.00	1.00	-	-	-	1.00	1.00	1.00
	III	1.00	1.00	1.00	-	-	-	0.98	1.00	0.99
Accuracy				1.00			-			0.99
Decision Tree	I	1.00	0.98	0.99	1.00	0.98	0.99	0.98	0.98	0.98
	II	1.00	1.00	1.00	1.00	1.00	1.00	1.00	1.00	1.00
	III	0.98	1.00	0.99	0.98	1.00	0.99	0.98	0.98	0.98
Accuracy				0.99			0.99			0.99
K – Nearst Neighbors	I	0.77	0.72	0.75	1.00	1.00	1.00	1.00	0.98	0.99
	II	0.90	0.95	0.92	1.00	1.00	1.00	1.00	1.00	1.00
	III	0.86	0.86	0.86	1.00	1.00	1.00	0.98	1.00	0.99
Accuracy				0.85			1.00			0.99
Support Vector Machine	I	0.96	0.96	0.96	1.00	0.98	0.99	1.00	0.96	0.98
	II	0.99	0.99	0.99	1.00	1.00	1.00	1.00	1.00	1.00
	III	0.98	0.98	0.98	0.98	1.00	0.99	0.97	1.00	0.98
Accuracy				0.98			0.99			0.99
Gaussian Naive Bayes	I	0.83	0.88	0.85	1.00	0.91	0.95	1.00	0.91	0.95
	II	0.97	0.96	0.97	0.96	1.00	0.98	0.97	1.00	0.99
	III	0.91	0.88	0.90	0.97	1.00	0.98	0.95	1.00	0.98
Accuracy				0.91			0.97			0.97
Multi Class Logistic Regression	I	0.96	0.96	0.96	1.00	0.93	0.96	1.00	0.91	0.95
	II	1.00	0.97	0.99	0.97	1.00	0.99	0.97	1.00	0.99
	III	0.97	1.00	0.98	0.97	1.00	0.98	0.95	1.00	0.98
Accuracy				0.98			0.98			0.97

### Machine-learning parameters are important according to the most accurate general machine-learning model

In the next section, the importance of each input parameter in the most accurate model, i.e., the random forest model, which included 24 cephalometric parameters as well as sex and age, was examined. The results shown in Table 3 demonstrate that the essential variable contributing to the model was Calculated_ANB (0.429), followed by the parameters’ ANB, Wits appraisal, SNB, SNPg and − 1/ML, respectively.

**Table 3:** Importance of the 24 cephalometric parameters and the covariates sex and age to classify an individual as skeletal class I, II, or III in the random forest machine-learning model

Variable	Importance
Calculated_ANB	0.429
ANB	0.164
Wits	0.131
SNB	0.045
SNPg	0.042
-1/ML	0.024
Gonial angle	0.020
ANBind	0.014
+1/NA (mm)	0.012
+1/SN	0.012
+1/NA	0.012
ML-NSL	0.011
SNA	0.009
NL/ML	0.009
PFH/AFH	0.009
Go-Me (mm)	0.008
age	0.007
facial Axis	0.006
-1/NB	0.006
SN-Ba	0.005
+1/NL	0.005
-1/NB (mm)	0.005
interincisal angle	0.005
S-N (mm)	0.005
NL/NSL	0.004
Gender	0.001

### Calculated_ANB as a single predictor

**Fig. 1:**
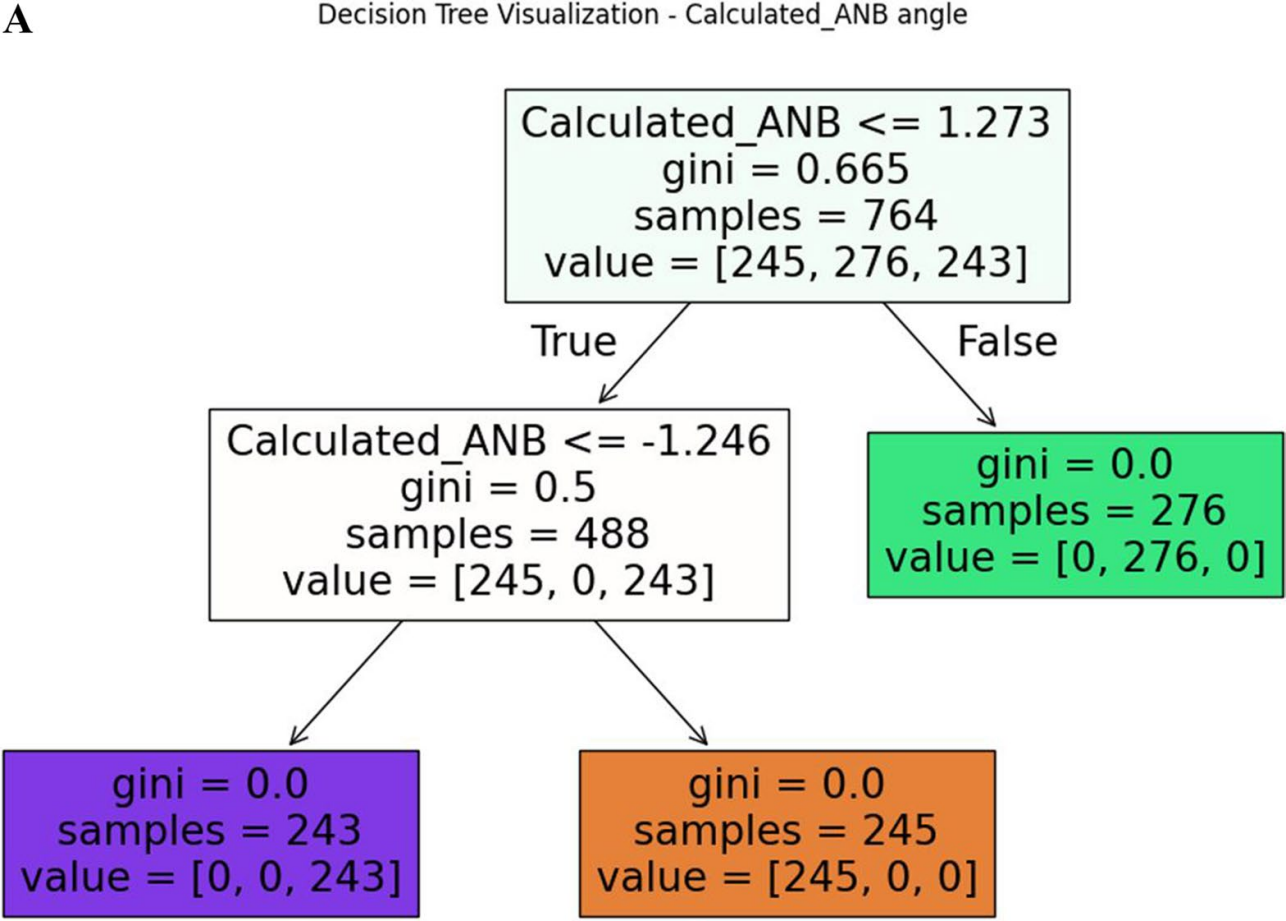

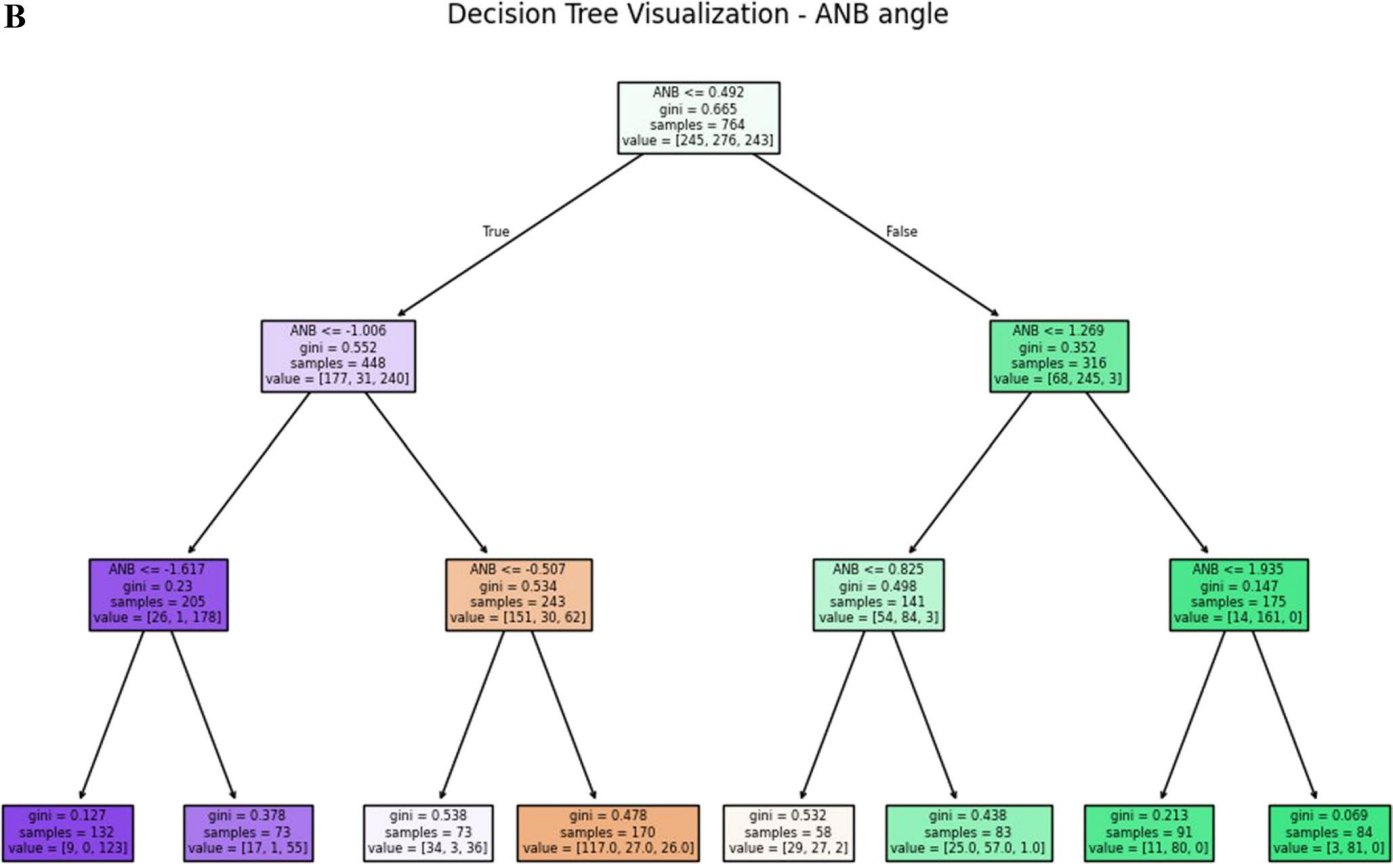
**A** presents the decision tree for the Calculated_ANB. Each node is labelled with the value of Calculated_ANB. In addition, in each node, the number of patients in each class is presented (samples) as well as the Gini score of purity (between 0 to 1). **B** presents the decision tree for the ANB angle

According to the previous section, Calculated_ANB was the most important variable with a score of 0.429, and remarkably higher than the subsequent parameters. Therefore, in this section machine-learning models with Calculated_ANB as the only predictor were applied. In all models, accuracy was 97% or higher. For example, the k-nearest neighbours model was able to classify the patients with 100% accuracy, and perfect precision, recall and F1 scores. In addition, the decision tree model was able to classify the patients with 99% accuracy, with perfect precision among skeletal class I, and II, and 98% precision among skeletal class III patients (Table 2). To understand the decision tree limits for each class, the decision tree for Calculated_ANB is visualised in Fig. 1A. The decision tree demonstrates that patients were categorised as skeletal class II, when Calculated_ANB was greater than 1.27. Besides, the patients were categorized as skeletal class I if Calculated_ANB ranged between − 1.25 to + 1.27 and as skeletal class III if Calculated_ANB was smaller than − 1.25. The Gini index, which ranges from 0 to 1 and describes the inequality among a distribution, clarifies that along the decision tree, equality of the (remaining) sample increased.

### Machine-learning models based on the parameters that define the Calculated_ANB (SNA, SNB, ML-NSL)

In this section, we applied machine-learning models based on the parameters needed to determine the Calculated_ANB, i.e., SNA, SNB, and ML-NSL. The results show that all models could classify the patients with at least 97% accuracy. The best models were random forest, decision tree, k-nearest neighbors, and support vector machine models with 99% accuracy. The full results of all models are described in Table 2.

### Can we gain accurate classification from the ANB angle (i.e., without the Calculated_ANB)? (Stepwise forward machine-learning models)

In the previous section, it was shown that the Calculated_ANB could predict an individual’s skeletal class precisely. This section aimed to examine machine-learning models, which do not include Calculated_ANB as an input parameter. Therefore, the importances of the input variables from the general model (Table 3) were used to perform a forward models’ system, which adds one more variable every time according to their importance. The results are presented in Table 4A-B. The results show that when including ANB angle only, the accuracy ranged between 71% in the decision tree model and 76% in the Gaussian naïve Bayes. The ANB angle decision tree, which is visualised in Fig. 1B, demonstrates the decision tree limits for each class. The decision tree faced difficulty in categorising the patients, which is also represented by the higher Gini scores in the lower nodes of the tree. In the next level, the parameter Wits appraisal was added to the ANB angle before applying the same machine-learning models. Here, accuracy ranged from 79% in the decision tree model to 82% in the model’s linear discriminant analysis and Gaussian naïve Bayes. The model’s accuracy improved by continuing the step-forward process and adding the next important parameter each time. This process was stopped when accuracy reached 92% in the model decision tree, support vector machine, and multi-class logistic regression with the input variables ANB angle, Wits appraisal, SNB, SNPg, −1/ML, and Gonial angle.

**Table 4:** A-B: Precision, recall, F1 score, and accuracy of the machine-learning model's Linear discriminant analysis, random forest, decision tree, K-nearest neighbours, support vector machine, Gaussian naïve Bayes, and multi-class logistic regression

**A**													
		Included parameters: ANB angle			Included parameters: ANB angle and Wits appraisal			Included parameters: ANB angle, Wits appraisal and SNB angle			Included parameters: ANB angle, Wits appraisal, SNB and SNPg angles		
	Class	Precision	Recall	F1 score	Precision	Recall	F1 score	Precision	Recall	F1 score	Precision	Recall	F1 score
Linear Discriminant Analysis	1	0.56	0.54	0.55	0.69	0.74	0.71	0.76	0.74	0.75	0.74	0.79	0.76
	2	0.79	0.83	0.81	0.88	0.84	0.86	0.86	0.91	0.88	0.88	0.88	0.88
	3	0.84	0.81	0.83	0.90	0.88	0.89	0.93	0.90	0.91	0.95	0.88	0.91
Accuracy				**0.74**			**0.82**			**0.85**			**0.85**
Random Forest	1	-	-	-	-	-	-	0.75	0.70	0.73	0.76	0.77	0.77
	2	-	-	-	-	-	-	0.87	0.91	0.89	0.90	0.91	0.90
	3	-	-	-	-	-	-	0.88	0.90	0.89	0.91	0.88	0.90
Accuracy				**-**			**-**			**0.84**			**0.86**
Decision Tree	1	0.52	0.49	0.50	0.64	0.68	0.66	0.72	0.63	0.67	0.75	0.74	0.74
	2	0.83	0.75	0.79	0.88	0.80	0.84	0.82	0.89	0.86	0.86	0.92	0.89
	3	0.75	0.88	0.81	0.84	0.88	0.86	0.90	0.90	0.90	0.93	0.86	0.89
Accuracy				**0.71**			**0.79**			**0.82**			**0.85**
K– Nearst Neighbors	1	0.56	0.53	0.54	0.66	0.58	0.62	0.73	0.70	0.71	0.75	0.74	0.74
	2	0.79	0.79	0.79	0.86	0.83	0.85	0.87	0.88	0.88	0.89	0.89	0.89
	3	0.82	0.86	0.84	0.80	0.93	0.86	0.88	0.90	0.89	0.88	0.90	0.89
Accuracy				**0.73**			**0.79**			**0.83**			**0.85**
Support Vector Machine	1	0.56	0.51	0.53	0.68	0.68	0.68	0.75	0.70	0.73	0.75	0.74	0.74
	2	0.79	0.83	0.81	0.88	0.84	0.86	0.86	0.91	0.88	0.88	0.89	0.89
	3	0.82	0.83	0.82	0.85	0.90	0.88	0.90	0.90	0.90	0.90	0.90	0.90
Accuracy				**0.73**			**0.81**			**0.84**			**0.85**
Gaussian Naive Bayes	1	0.58	0.63	0.61	0.69	0.74	0.71	0.75	0.75	0.75	0.77	0.77	0.77
	2	0.79	0.82	0.81	0.88	0.84	0.86	0.87	0.89	0.88	0.88	0.91	0.90
	3	0.90	0.80	0.85	0.90	0.88	0.89	0.93	0.90	0.91	0.93	0.90	0.91
Accuracy				**0.76**			**0.82**			**0.85**			**0.86**
Multi Class Logistic Regression	1	0.55	0.51	0.53	0.67	0.67	0.67	0.75	0.72	0.73	0.78	0.75	0.77
	2	0.79	0.83	0.81	0.88	0.84	0.86	0.87	0.89	0.88	0.90	0.92	0.91
	3	0.81	0.81	0.81	0.84	0.88	0.86	0.90	0.90	0.90	0.90	0.90	0.90
Accuracy				**0.73**			**0.80**			**0.84**			**0.86**
**B**													
		Included parameters: ANB angle, Wits appraisal, SNB, SNPg and -1/ML				Included parameters: ANB angle, Wits appraisal, SNB, SNPg, -1/ML and Gonial angle							
	Class	Precision		Recall	F1 score	Precision	Recall	F1 score					
Linear Discriminant Analysis	1	0.81		0.88	0.84	0.80	0.89	0.84					
	2	0.91		0.95	0.93	0.94	0.95	0.94					
	3	1.00		0.86	0.93	0.98	0.85	0.91					
Accuracy					**0.90**			**0.90**					
Random Forest	1	0.82		0.88	0.85	0.87	0.85	0.85					
	2	0.91		0.95	0.93	0.91	0.95	0.93					
	3	1.00		0.88	0.94	0.95	0.95	0.95					
Accuracy					**0.91**			**0.91**					
Decision Tree	1	0.78		0.88	0.83	0.86	0.88	0.87					
	2	0.91		0.95	0.93	0.93	0.93	0.93					
	3	1.00		0.83	0.91	0.97	0.95	0.96					
Accuracy					**0.89**			**0.92**					
K– Nearst Neighbors	1	0.81		0.82	0.82	0.82	0.86	0.84					
	2	0.89		0.92	0.90	0.92	0.95	0.94					
	3	0.98		0.92	0.95	0.96	0.88	0.92					
Accuracy					**0.89**			**0.90**					
Support Vector Machine	1	0.87		0.84	0.86	0.85	0.88	0.86					
	2	0.91		0.95	0.93	0.94	0.95	0.94					
	3	0.97		0.95	0.96	0.96	0.92	0.94					
Accuracy					**0.92**			**0.92**					
Gaussian Naive Bayes	1	0.86		0.88	0.87	0.83	0.88	0.85					
	2	0.91		0.96	0.94	0.94	0.95	0.94					
	3	1.00		0.92	0.96	0.96	0.90	0.93					
Accuracy					**0.92**			**0.91**					
Multi Class Logistic Regression	1	0.85		0.79	0.82	0.86	0.88	0.87					
	2	0.89		0.95	0.92	0.95	0.95	0.95					
	3	0.95		0.93	0.94	0.95	0.93	0.94					
Accuracy					**0.90**			**0.92**					

### Deep-learning algorithms (ANN)

#### General deep-learning model

**Fig. 2:**
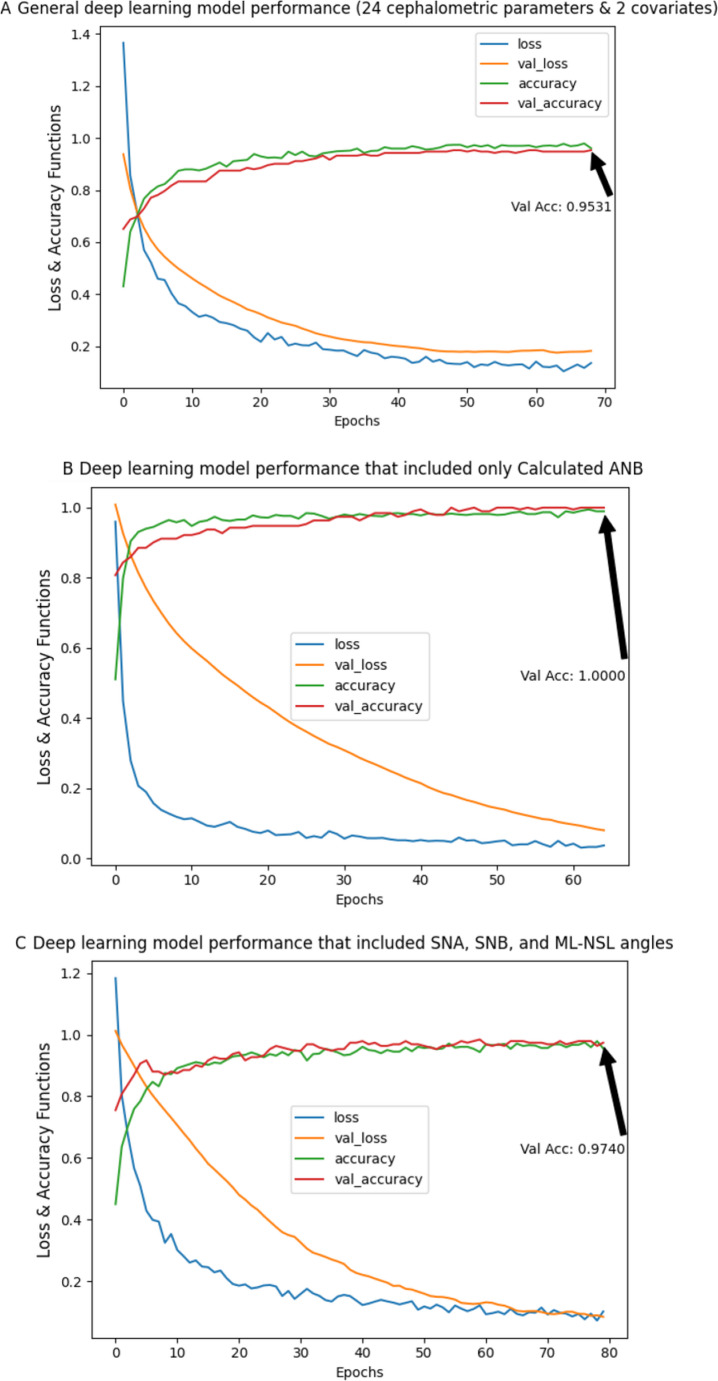
Performance of the deep-learning models to classify the patients as skeletal class I, II or III, including training loss function, validation loss, training accuracy and validation accuracy. In (**A**) the model includes all cephalometric variables, sex and age. In (**B**) the model is based on Calculated_ANB only. **C** shows the performance of model based on the variables SNA, SNB and ML-NSL.

A general deep-learning model, an ANN, which included all cephalometric parameters as well as the covariates sex and age, was performed. The results demonstrate the ability of this model to predict an individual’s skeletal class with an accuracy of 95.31% in the validation data (training-accuracy = 98.17%, validation-accuracy = 95.31%). The performance of the model is presented in Fig. 2A.

#### The deep-learning model that included Calculated_ANB as a single predictor

According to the results of the importance of input variables for the machine-learning model random forest, Calculated_ANB was the most crucial variable. The results of the deep-learning model, which included only Calculated_ANB as a predictor, demonstrate 100% validation accuracy (training-accuracy = 98.42%, validation-accuracy = 100%) (Fig. 2B).

#### Deep-learning models based on the parameters needed for the Calculated_ANB (SNA, SNB, and ML-NSL)

In the following model, the parameters, which are required to determine Calculated_ANB, i.e., the angles SNA, SNB and ML-NSL, were included. The model’s validation-accuracy was 97.40% (training-accuracy = 96.55%, validation-accuracy = 97.40%) (Fig. 2C).

#### ANB measured angle power to accurately classify orthodontic patients (Stepwise forward machine-learning models)

In the previous sections, the results showed a high accuracy when incorporating the Calculated_ANB in the deep-learning models. In addition, almost similar accuracy was achieved when using the parameters incorporated in the Calculated_ANB. In contrast, the results revealed insufficient accuracy of deep-learning models based on the ANB angle only. Furthermore, the addition of the next important variables, according to the random forest model presented in Table 3, did not significantly improve the accuracy of the models.

#### The use of different classifiers

**Fig. 3:**
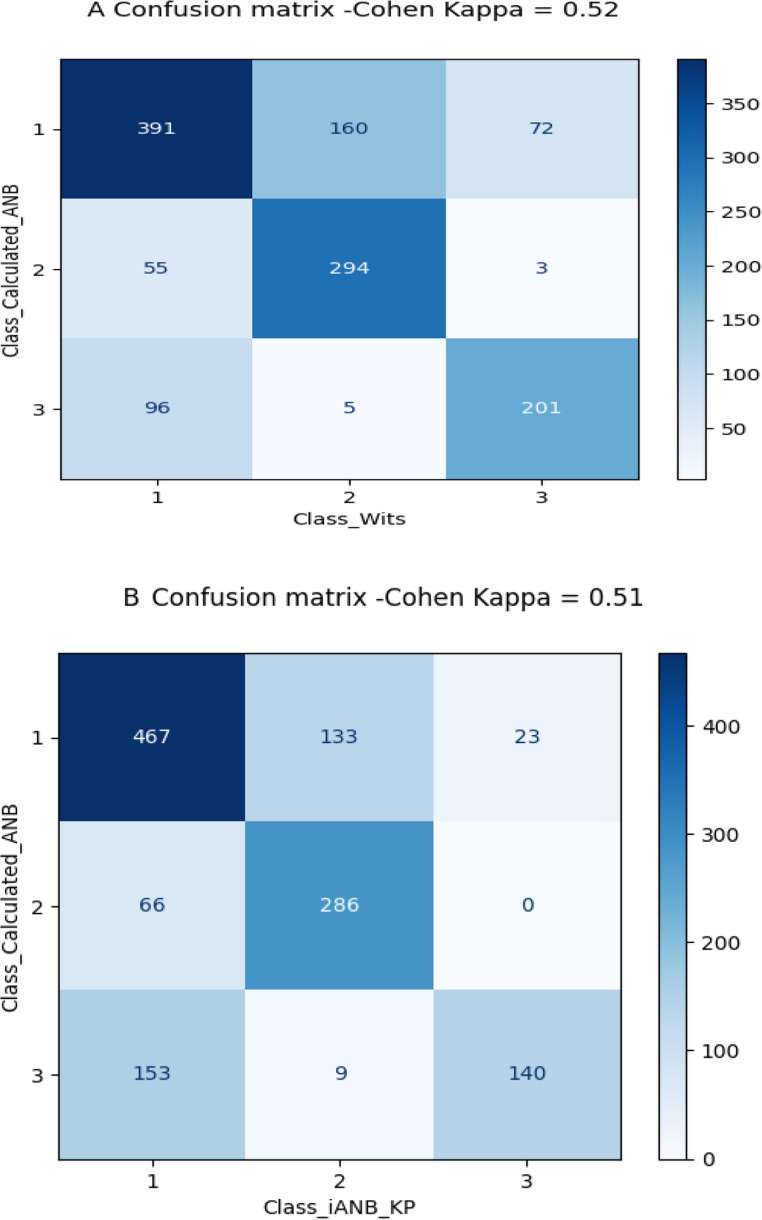
Confusion matrices illustrating the agreement between the gold standard in diagnosing skeletal class (i.e., Calculated_ANB) and other classification methods- the Wits appraisal (**A**), and iANB_KP (**B**). Furthermore, Cohen Kappa describes the agreement between the methods included in each figure

In this study, Calculated_ANB was used as the gold standard to classify patients as skeletal class I, II and III. The agreement between the gold standard and other classifying methods was visualised applying a confusion matrix and Cohen’s Kappa. The agreement between the methods Calculated_ANB and Wits appraisal was moderate, with a Kappa score of 0.52. The confusion matrix in Fig. 3A illustrates that 391 (*n* = 391, 62.76%) patients were diagnosed as skeletal class I by both methods. 294 (*n* = 294, 83.2%) cases were diagnosed as skeletal class II, and 201 (*n* = 201, 66.55%) patients were classified as skeletal class III by both methods. Finally, the agreement between the methods Calculated_ANB and iANB_KP was 0.51. Here, the confusion matrix in Fig. 3B demonstrates that 467 (*n* = 467, 74.95%) patients were diagnosed as skeletal class I, and 286 (*n* = 286, 81.25%) individuals were classified as skeletal class II by both methods. In contrast, only 140 (*n* = 140, 46.35%) patients were diagnosed as skeletal class III by these two methods.

#### The performance of machine-learning & deep-learning algorithms based on other classification methods

The same machine-learning and deep-learning algorithms described above were repeated using the sample’s classification with the Wits appraisal. In the general models, which included all parameters, the random forest and decision tree models could classify the patients with 100% accuracy. Comparing the importance of all input variables in the random forest model, Wits appraisal was the most critical parameter, followed by Calculated_ANB, ANB, and − 1/ML, respectively (Table 5). Including only the most important input parameter, i.e. Wits appraisal, resulted in 100% accuracy in the decision tree and k-nearest neighbours models. Regarding the deep-learning models (ANN) based on this classification method, the general deep-learning model, which was based on all parameters, achieved a validation-accuracy of 94.09% (training-accuracy = 98.09%, validation-accuracy = 94.09%). The deep-learning models, which considered Wits appraisal only, resulted in a higher validation accuracy, being 99.51% (training-accuracy = 99.04%, validation-accuracy = 99.51%).

**Table 5:** Importance of the 24 cephalometric parameters and the covariates sex and age to classify an individual as skeletal class I, II, or III in the random forest machine-learning model. This model was based on a sample, which was classified according to the wits appraisal

Variable	Importance
Wits appraisal	0.53
Calculated_ANB	0.13
ANB	0.08
−1/ML	0.02
SNB	0.02
SNPg	0.02
+ 1/NA (mm)	0.02
Gonial angle	0.01
PFH/AFH	0.01
+ 1/NA	0.01
Age	0.01
SNA	0.01
Go-Me (mm)	0.01
ANB indiv	0.01
+ 1/SN	0.01
ML-NSL	0.01
NL/NSL	0.01
S-N (mm)	0.01
−1/NB	0.01
−1/NB (mm)	0.01
Facial axis	0.01
NL/ML	0.01
SN-Ba	0.01
+ 1/NL	0.01
Interincisal angle	0.01
Sex	0.00

## Discussion

This prospective cross-sectional study aimed to develop both a machine-learning and a deep-learning model to correctly classify German orthodontic patients of all ages and sexes as skeletal class I, II or III. The machine- and deep-learning models, including all input parameters, achieved 100% and 95.31% accuracy, respectively. Secondary outcomes included the comparison of different classification methods (Wits appraisal, iANB_KP) as well as the repetition of the AI-methods for an alternative classification of the sample (Wits appraisal). Here, the general models of the machine- and deep-learning with all input variables resulted in an accuracy of 100% and 94.09%, respectively.

The study population was inhomogenously distributed with respect to skeletal class I, II and III, but representative of the German patients at the study centres. Based on Calculated_ANB, the majority of the participants had skeletal class I (48.79%), whereas 27.56% and 23.64% presented a class II and III, respectively. Depending on the population assessed, these distributions of skeletal class vary. For example, in a group of primarily Italian children skeletal class I was present in 49.14%, similar to skeletal class II (47.43%), whereas skeletal class III was found only in 3.43% [27]. Although a variety of classification methods is described in the literature, the primary outcome of this study was based on classifying the participants according to the Calculated_ANB, as reported by Panagiotidis and Witt [7]. This method was chosen due to increased precision in diagnostics [5, 7] and because of the ability to compare it with other publications [20]. Among all skeletal classes, females slightly dominated, but not to a clinically relevant extent (< 7%). According to the mean age of 13 and 14 years in class I and II, and III, respectively, most patients were adolescents. The patients in this investigations were younger than the Arab cohort of class II and III patients with mean ages of 17 and 18 years, respectively [20].

Various machine-learning models were performed with partly variable performances. When all input parameters were included, i.e. all cephalometric variables as well as sex and age, the RF model performed best with 100% accuracy, and 100% precision, recall and F1 scores in each class. This accuracy was clearly higher than the 87% accuracy achieved in machine-learning models for skeletal class I and II diagnosis in Arabs [21], but comparable to the 99% accuracy for skeletal class II and III classification in Arab patients [20]. Within the general model, the most important parameter was Calculated_ANB, followed by ANB, Wits appraisal, SNB, SNPg and − 1/ML, respectively. Interestingly, all of these skeletal parameters are sagittal ones, as is skeletal class. It appears, that the sagittal parameters of the mandible (SNB, SNPg) have a bigger impact on the automated classification than those of the maxilla. Among the most crucial variables, there is one dentoalveolar parameter, −1/ML. The importance of the inclination of the lower incisors’ could be explained by its relation with skeletal class, which is expressed in floating norms for the inclination and position of these teeth [28, 29].

The machine-learning models, based on Calculated_ANB only, resulted in 97–100% accuracy. Within the model DT, the lower and upper limits of Calculated_ANB for skeletal class I were − 1.25 ° and + 1.27 °, respectively. Compared to the manually applied limits in the gold standard (± 1.5 °) the AI-method used slightly narrower limits to achieve the high accuracy of 99% and final Gini scores of 0, indicating perfect equality of the automatically classified groups. Increasing the number of input variables by considering all those needed to determine Calculated_ANB, led to slightly less accuracy, ranging between 97 and 99%. In a work that was done by Suryakumar et al. [30] about the critical dimension in data mining, and demonstrated that shown that the phenomenon of critical dimension indeed exists for many datasets, and in each dataset was found that the elimination of irrelevant feature can gain a higher accuracy. In contrast, including ANB only, resulted in clearly lower accuracy (71–76%), which identifies this model as inappropriate to determine an individual’s skeletal class. This observation could be explained by the imprecise diagnosis of this parameter in case the maxillary prognathism (SNA) and/or mandible’s inclination deviate from the empirical norm values [7]. Hence, from our results, it can be recommended to include Calculated_ANB in the machine-learning model to avoid a (clearly) noticeable loss of accuracy.

The general deep-learning model (ANN) presented almost perfect accuracy during validation (95.31%), which was even better (100% validation-accuracy) with Calculated_ANB only. This accuracy was comparable with the mean-accuracies of 90.50–95.70% reported for the CNN-models of Yu et al. [31]. Similar to the machine-learning model, ANB as a single predictor achieved insufficient accuracy in the deep-learning algorithm. The advantage of the deep-learning model compared to the machine-learning algorithm is.

Comparing the gold standard of this study, Calculated_ANB, with two other classification methods, Wits appraisal and iANB_KP, resulted only in moderate accuracy (Kappa 0.52 and 0.51, respectively). This clarifies the need to carefully look at the classification method chosen, when comparing the data with other publications. For example, Nan et al. used a combination of ANB and Wits appraisal [18], which could have led to a different distribution within their study collective. The advantage of iANB_KP [5] is the update for a contemporary population compared to Calculated_ANB [7]. Wits appraisal is based on a linear measurement and frequently used [6, 18, 31], but does not present an individualised norm, which could potentially have a negative impact on the precision. AI models based on the sample’s classification according to the Wits appraisal resulted in perfect accuracy (100%) in the general model with complete input variables as well as with Wits appraisal, the most important parameter in this model only. This observation is identical to the 100% accuracy reported for Calculated_ANB-classified samples. Also comparable results were reached with the deep-learning models, which considered all variables (validation-accuracy 94.09%) or Wits appraisal only (validation-accuracy 99.51%).

### Limitations

The limits chosen to differentiate between skeletal class I, II, and III (1.5 °) were slightly different from those suggested by Panagiotidis and Witt (1 °) [7], which should be considered during comparison with other studies. This definition was applied to exclude borderline cases from the skeletal classes II and III groups. Another limitation is the missing assessment of patients’ ethnicity. However, since only German study centers were used for recruitment, it appears unlikely that many patients presented different ethnic groups. Furthermore, the study size of 1277 patients should be increased in future investigations, allowing for validation of the AI algorithms presented even in a bigger population. Finally, the cephalometric analysis used as the gold standard could be incorrect. But, interrater and intrarater reliability testing performed revealed reproducible measurements.

## Conclusion

Machine- and deep-learning models were successfully developed for classifying German orthodontic patients as skeletal class I, II and III. These findings could be implemented in a digital and automated workflow to relieve the orthodontic practitioner, and integrated along with the available tools and clinical experience, for a fast, efficient and accurate diagnosis. Depending on the gold standard applied to classify the data pool, few cephalometric parameters are especially crucial for automated classification, which clarifies the need for precise cephalometric analyses. Future studies should aim to increase the study size and validate the data in bigger cohorts. Besides, a user-friendly application that will be based on the models presented in this study, should be examined in a real-world orthodontist’s clinic, while adjusting these applications to better help the orthodontists’ preferences and needs.

## Supplementary Information

Below is the link to the electronic supplementary material.

ESM 1 (PDF 201 KB)

## Data Availability

Data can be accessed via the corresponding author.
