# Supplementary material for: Automated classification of skeletal malocclusion in German orthodontic patients
Source: Clin Oral Investig. 2025 Aug 5;29(8):396. doi: 10.1007/s00784-025-06485-0 (PMC12325434; doi:10.1007/s00784-025-06485-0)
Supplement: Supplementary file 1 — (PDF 201 KB) [file 784_2025_6485_MOESM1_ESM.pdf]

Supplementary Table 1. Cephalometric parameters definitions – this table represents the cephalometric parameters that were extracted from the lateral cephalogram image, and were included in the AI models

**Dimension/Group**	**Parameter**	**Unit**	**Definition**
Vertical Analysis	NL/ML (anatomic)	°	The angle between the NL and ML
Vertical Analysis	SNL/ML (anatomic)	°	The inclination of the mandible (mandibular inclination) relative to the nasion-sella line (anterior skull base, SNL)
Vertical Analysis	NL/NSL	°	The angle between Sella-Nasion-line (NSL = SN) and nasal line (Spa-Spp)
Vertical Analysis	PFH/AFH	%	The ratio between posterior (SGo) and anterior (NMe) facial height
Vertical Analysis	Gonial Angle	°	The angle between ML and line GoAr at Gonion
Vertical Analysis	Facial axis	°	The angle between the lines NBa and PtGN'
Sagittal Analysis	Angle SNA	°	The angle between Sella, Nasion, and point A
Sagittal Analysis	Angle SNB	°	The angle between Sella, Nasion, and point B
Sagittal Analysis	ANB	°	The angle between Nasion, point A, and point B
Sagittal Analysis	ANB_{ind}	°	ANB_{ind} = (−35.16 + 0.4 · SNA + 0.2 · ML-NSL) according to Panagiotidis and Witt
Sagittal Analysis	Calculated_ANB		ANB - ANB_{ind}
Sagittal Analysis	SN-Ba	°	Central saddle angle. It describes the extent of the skull base flexion
Sagittal Analysis	SNPg	°	The angle between Sella, Nasion and Pogonion
Sagittal Analysis	S-N	mm	The S-N line represents the anterior cranial base. It is constructed by connecting the points sella turcica and the Nasion
Sagittal Analysis	Go-Me	mm	The mandibular plane as a line connecting the points gonion and menton
Sagittal Analysis	Wits	mm	This parameter measures the extent to which the jaws are related to each other anteroposteriorly.
Growth Analysis	ML-NSL	°	The angle formed between the ML and NSL lines
Dental Analysis	(+1/NL)	°	The angle between upper incisors' tooth axis and line NL
Dental Analysis	(+1/SN)	°	The angle between upper incisors' tooth axis and line SN
Dental Analysis	+1/NA	°	The angle between upper incisors' tooth axis and line NA
Dental Analysis	+1/NA	mm	The perpendicular distance between the upper central incisor to N-A provides information about the sagittal position of the incisor teeth.
Dental Analysis	-1/ML (anatomic)	°	The relative anteroposterior angulation of the lower incisor teeth is determined by relating the most protruding incisor tooth to the mandibular plane (ML)
Dental Analysis	(-1/NB)	°	The lower central incisor to N-B indicates the axial inclination of these teeth
Dental Analysis	(-1/NB)	mm	The perpendicular distance between the lower central incisor to N-B provides information about the sagittal position of the incisor teeth.
Dental Analysis	Interincisal angle	°	The inter-incisal angle relates the inclination of the upper incisor to that of the lower incisor

Supplementary Figure 1. Lateral cephalogram parameters

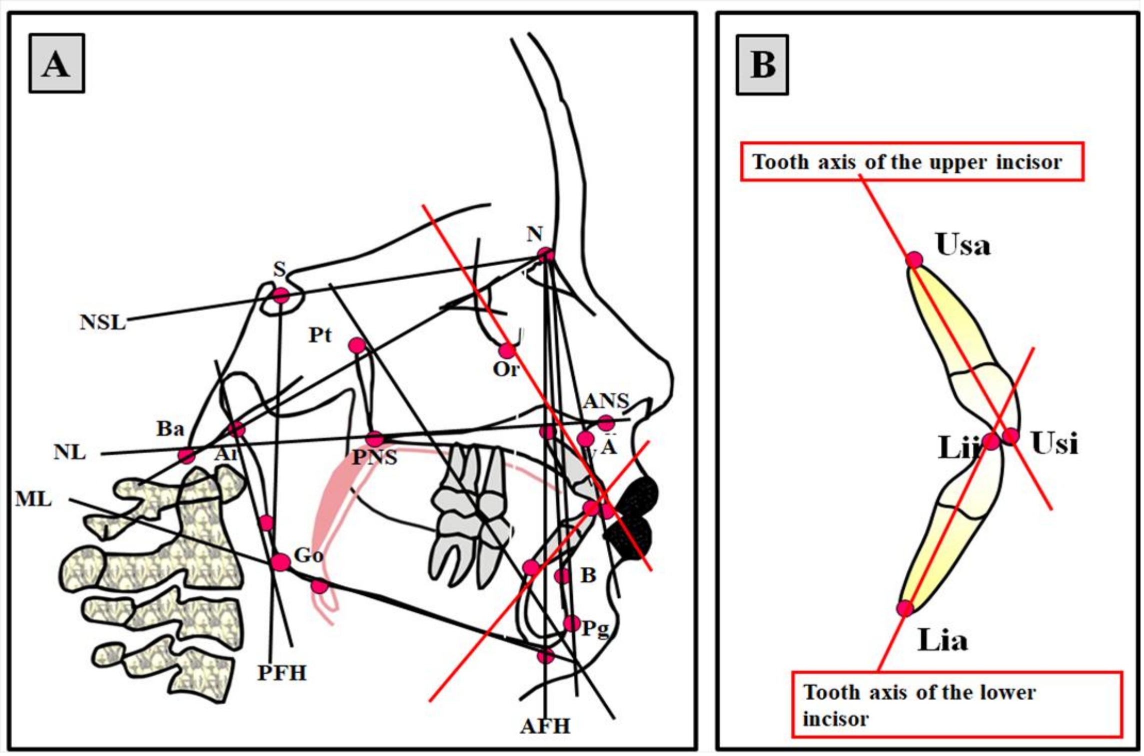

Appendix Figure 1: Represents the most crucial points extracted from the lateral cephalogram in this study (**Figure 1A**). Among these points are Nasion (N), Sella (S), Pterygoid point (Pt), Basion (Ba), Orbitale (Or), Subspinale ("A" Point), Supramentale ("B" point), and other points. **Figure 1B** shows the tooth axis of the upper and lower incisors positions and angles.
